# The survival and clinical performance of anterior composite resin restorations and posterior indirect and cast restorations used to treat generalised tooth wear

**DOI:** 10.1038/s41415-024-7617-z

**Published:** 2024-08-09

**Authors:** Sachin Shah, Kenneth Hemmings, Akil Gulamali, Aviva Petrie, Junaid Saleem Malik

**Affiliations:** 41415117313001 Registered Specialist in Prosthodontics, Eastbourne, UK; 41415117313002 Consultant in Restorative Dentistry and Honorary Clinical Associate Professor, Eastman Dental Hospital and Institute, University College London and Hospital, UK; 41415117313003 Registered Specialist in Prosthodontics, Surrey, UK; 41415117313004 Honorary Associate Professor, Eastman Dental Institute, University College London, UK; 41415117313005 NIHR Academic Clinical Fellow in Prosthodontics, UCL Eastman Dental Institute, UK; Speciality Registrar in Prosthodontics, University College London and Hospital, UK; Associate Clinical Lecturer, UCL Eastman Dental Institute, UK

## Abstract

**Objective** To evaluate the survival and clinical performance of restorative materials used in the rehabilitation of generalised severe tooth wear within a UK NHS postgraduate teaching hospital.

**Methods** The clinical performance of 527 restorations on 20 patients with generalised severe tooth wear was reviewed after a mean period of five years. Anterior teeth were restored with direct composite resin and posterior teeth with indirect restorations. The study used the modified United States Public Health Service criteria for restoration assessment. Survival of the restorations was analysed using Kaplan-Meier survival curves, the log-rank test and the Cox proportional hazards regression analysis.

**Results** The sample included 20 participants: 13 men and 7 women, with a median age of 51.8 years (range: 33-73 years). The median survival time for all restorations was 11.3 years when major failures were considered and 5.9 years for restorations when all types of failure were considered. A median survival time of 5.9 years for composite resin restorations and over seven years for cast restorations was found when considering all failures. Composite resin restorations commonly failed as a result of fracture, wear and marginal discolouration. Factors significantly influencing restoration survival were the material used, aetiology, incisal relationship and tooth location. The biological complications associated with this treatment regime were rare. Patient satisfaction remained generally high, with greatest dissatisfaction related to treatment time.

**Conclusions** The use of anterior composite resin with posterior indirect restorations to treat generalised severe tooth wear is a viable treatment modality with very few major complications.

## Key points

- The article aims to demystify the appropriate use of restorative materials in the management of generalised tooth wear.
- The manuscript will help clinicians make more appropriate choices of restorative materials based on their long-term performance as highlighted in this paper.
- The manuscript will help guide clinicians on the appropriateness of splint therapy in the management of tooth wear.

## Introduction

The progression of tooth wear results in a reduction in tooth volume, most commonly as a reduction in clinical crown height. This is frequently compensated by eruption of the teeth, which can be is variable. Setchell^1^ described compensation as a mechanism that advances the teeth into the ‘occlusal interface' to maintain functional contacts as wear occurs. The occlusal vertical dimension (OVD) does not appear to decrease and the lower third face height remains unaffected in the majority of dentate patients.

As compensation occurs, the attachment apparatus remodels with slow tooth movement, often leaving the mucogingival line in its original position and producing a widened zone of attached mucosa. The clinical crowns are shorter, the roots of teeth tend to taper, and the tissues overlying the roots may appear thick. Mesial drift can occur due to interproximal wear and fine positional changes of teeth may occur to maintain the functional capacity as wear progresses.

In pathological tooth wear, these compensatory mechanisms will usually occur with time and the restoration of teeth can be compromised by reduced tooth structure and the lack of available interocclusal space. If unmanaged, it can be of concern to patients and threaten tooth survival. The decision to treat and restore worn teeth can be very subjective, often based on clinical judgement or experience of the treating clinician and the presenting concerns of the patient.

The findings from a review by Mesko *et al.*^2^ showed that although the rehabilitation of severely worn teeth is common, there appears to be no strong published evidence to support the use of a specific material or technique.

The choice of material to be used for the restoration could be crucial. Studies on the wear process affecting restorative materials are almost exclusively experimental laboratory trials, and extrapolating these results to the clinical situation is very difficult.^3^

It is very important to consider factors that influence the wear resistance of natural and restored teeth. Besides the risk of mechanical failures under conditions of excessive load, biological failures are just as likely. In cases of high load conditions, metal or metal-ceramic restorations seem to be the safest choice but have a finite life span. The few clinical studies published on wear of materials in bruxist patients indicate only small differences in wear resistance of gold and ceramic materials, whereas resin-based materials showed three to four times larger substance loss.

It was clear from the Mesko *et al*. review^2^ that large variation in outcomes exist with annual failure rates varying from 0.7%^4^ to 26.3%^5^ for the performance of direct resin composites. From these studies, it was clear that microhybrid composite resins faired considerably better than the older microfilled resins.

There is a need for further evidence-based studies to facilitate treatment strategies for severely worn dentitions, with emphasis not only on restoration survival, but also a patient-centred approach, including tooth survival and patient satisfaction. The aim of this study was therefore to evaluate the long-term (ten years) survival and clinical performance of various restorative materials provided at an increased OVD used to manage generalised severe tooth wear.

## Method

### Sample

**Fig. 1:**
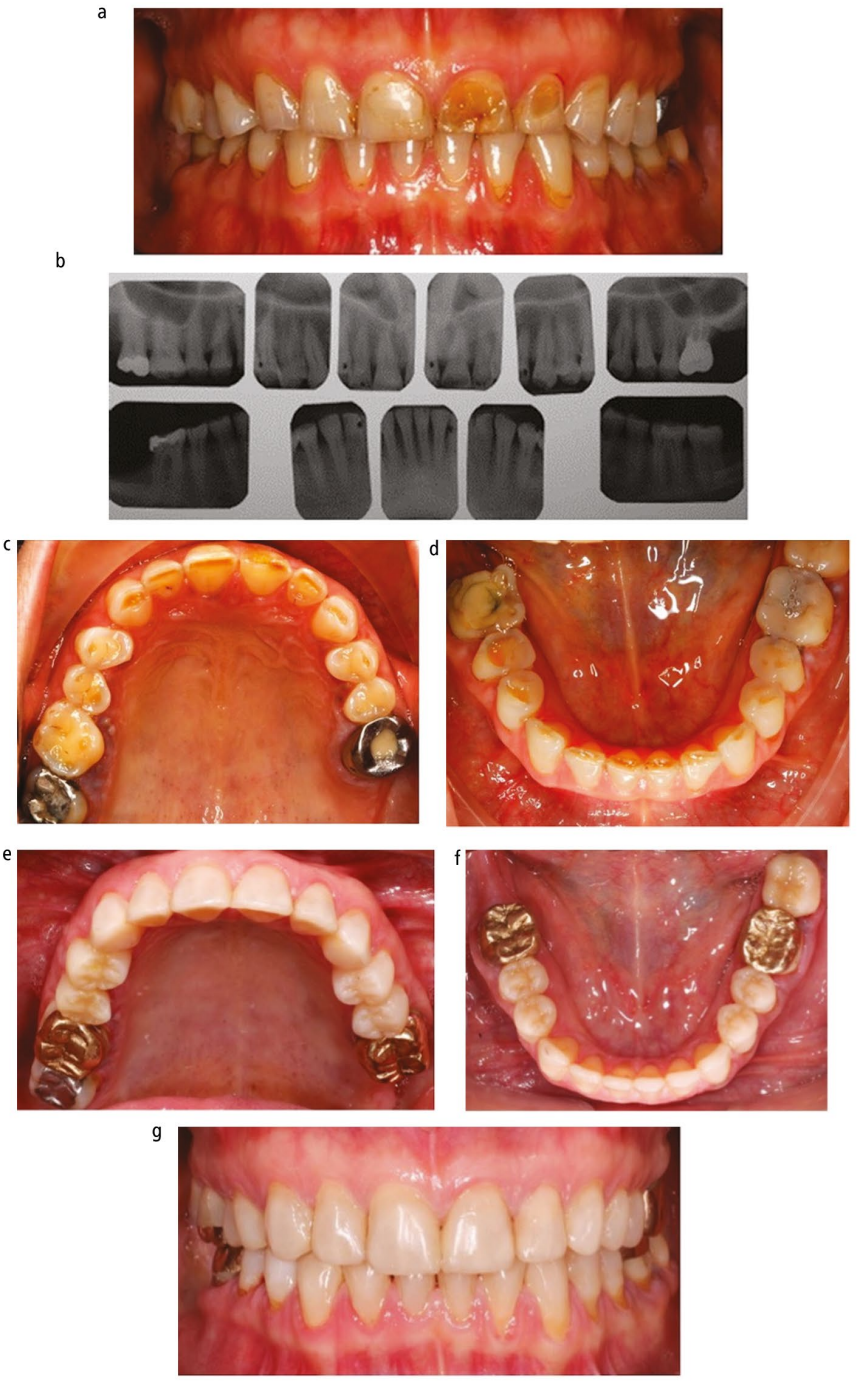
a) Pre-op anterior tooth wear and posterior failed restorations. b) Radiographs of case. c) Upper occlusal view. d) Lower occlusal view. e) Upper arch restored with anterior composites and posterior crowns at an increased OVD. f) Lower arch restored in a similar way. g) Final appearance. Minimal further damage to the dentition to restore appearance and function. Images courtesy of Motasum Abu-Awwad

The study was granted ethical approval by the NHS National Research Ethics Service, Cornwall & Plymouth Research Ethics Committee (15/SW/0040). The sample included a group of 20 patients (13 men and 7 women) treated for generalised tooth wear treated with anterior composite resins (incisors to canines) and posterior indirect restorations (premolars and molars) at an arbitrarily increased vertical dimension (Fig. 1). The age of patients ranged from 33-73 years old with a mean of 51.8 years. These patients were treated between April 1994 and April 2014 at the Eastman Dental Hospital with a mean follow-up of 62 months.

A total of 527 restorations were reviewed in the 20 patients. This included 298 composite restorations, 21 amalgam restorations, 172 cast restorations and 36 implant restorations placed, with a mean of 26.35 teeth restored per patient. All treating clinicians were postgraduate students or specialty registrars on a specialist training pathway.

### Collection of data

Data collection involved a 45-minute clinical assessment at the Department of Prosthodontics at Eastman Dental Hospital (EDH) with reference to clinical records; assessment of restorations using modified United States Public Health Services (USPHS) criteria; radiographic analysis; and statistical analysis following database collation of the clinical findings being carried out.

#### Clinical records

The subjects were identified and recruited from the patient allocation register held by the restorative department. Subsequent access to dental records was thus possible and enabled confirmation of a diagnosis and pertinent details about material selection. Dates of initial placement, repairs, or replacement was then transferred to the data collection *pro forma*. The clinical records, including some previous intra-oral photographs and study casts, served to rectify any discrepancies that became evident during data collection.

#### Recall examination

During the 45-minute recall, a thorough history and clinical examination was carried out for data collection. The following information was recorded:

- Patient details (study ID, date of birth, sex)
- Treatment details (treatment date, operator and aetiology of tooth wear)
- Restoration details (material used, opposing dentition and occlusal relationship)
- Restoration failure details were obtained from the patient, including history of repair or replacement, date, tooth involved and cause of failure.
- Restoration assessment (USPHS criteria scores)
- Patient satisfaction (based on existing restorations and willingness to have the same treatment again).

Clinical photographs and study casts were taken and compared to patients' previous clinical photographs and study casts if available. To decrease any variation in subjective assessment, intra- and inter-examiner training and calibration were undertaken.

Radiographic examination was not routinely carried out during this study but obtained when clinically indicated. Pre-operative radiographs were available for assessment within the clinical records.

#### Restoration assessment

Restorations were clinically assessed both directly and indirectly. Indirect observation utilised magnified digital images and study casts. The modified USPHS criteria were used to assess the restorations with eight criteria (anatomic form; marginal adaptation; wear; surface roughness; marginal discoloration; colour match; gingival health; and post-operative pain).^6^ Calibration of a single assessor revealed an intra-examiner Kappa score on agreement of 0.854 and the inter-examiner score of 0.813 which suggested substantial agreement, respectively.

#### Patient satisfaction

Patient satisfaction was assessed using a modified OHQoL-UK (UK Oral Health-Related Quality of Life) questionnaire.

The subjects' responses were recorded on a Likert scale, selecting one of five ratings per category: very good, good, none, bad and very bad.

An additional four questions were added at the end of the questionnaire to assess the participants' satisfaction on a range of aspects related to their treatment:

1. Satisfaction with improvement in dental condition following treatment
2. Satisfaction with the dental treatment and care they received at EDH
3. Satisfaction with the duration of dental treatment at EDH
4. Satisfaction with their dental appearance following dental treatment at EDH.

### Survival analysis

The collated data were entered on a spreadsheet using Statistical Programme for Social Sciences (SPSS 24). The objective of the data analysis was to assess the restoration survival function and clinical factors contributing to restoration survival during these treatment regimes. The variables tested were:

- Patient sex
- Operator
- Aetiology of tooth wear (Table 1)

**Table 1:** Distribution of tooth wear aetiology

Suspected aetiology	Number of patients
Primarily erosion	8
Primarily attrition	4
Combination	8
**Total**	**20**
- Material distribution (Table 2 and Table 3)

**Table 2:** Restorative material distribution anteroposterior

	Anterior	Canine	Posterior	Total
No restoration	8	4	3	15
Composite resin	150	63	85	298
Cast restoration	2	13	157	172
Implant restoration	0	0	36	36
Amalgam	0	0	21	21
**Total**	**160**	**79**	**301**	**542**

**Table 3:** Restorative material distribution between arches

	Maxilla	Mandible	Total
No restoration	1	14	15
Composite resin	155	143	298
Cast restoration	94	78	172
Implant restoration	10	26	36
Amalgam	9	12	21
**Total**	**269**	**273**	**542**
- Nature of opposing dentition (Table 4)

**Table 4:** Nature of opposing dentition

	Frequency	Percent
No opposing tooth	20	3.7
Unrestored tooth	15	2.8
Restored tooth	471	86.9
Implant restoration	36	6.6
**Total**	**542**	**100.0**
- Incisal relationship (Table 5).

**Table 5:** Number of restorations placed in different incisal relations

Incisal relation	Number of patients	Number of restorations
Class I	9	237
Class II Div 1	5	138
Class II Div 2	5	141
Class III	1	26
**Total**	**20**	**542**

The baseline date was taken as the date of restoration placement. Survival of a restoration was defined as the interval between date of placement and date of failure. The restorations that were repaired or replaced were recorded according to mode of failure and time to failure obtained from the patients. The restorations determined not to have failed were censored.

Survival analysis was carried out at two different levels of failure:

- Major failure - defined as any restorations that required complete replacement and included any recall restoration that had a USPHS score of three
- Combined major and minor failure - included all restorations in the major failure group, all past failures that had required repair or refinishing for any reason, and those that were placed in the USPHS score of two for any of the assessment criteria. Exception to this was when restorations were surviving and the only USPHS score of two was related to wear, the rest of the assessment criteria scoring a USPHS score of one. This methodology was consistent with the previous related study by Gulamali *et al.*^7^

Survival analysis was performed on each variable initially using the Kaplan-Meier approach to assess the effect of that variable on survival. Log rank values were calculated addressing the null hypothesis that there are no differences in survival times in the categories of variables being tested. Where the log rank test gave a value of p <0.05, the categories of variable differed significantly in their effect on the survival of the restorations. Where p <0.05 and the variable had more than two categories, the data were analysed in pairs to establish where the difference lay and to assist in calculating baseline hazards for the subsequent Cox proportional hazards model. This Cox proportional hazards regression analysis was used to evaluate the independent effects of the different variables included in the model on the survival of the restorations.

## Results

### Restorative material variables

#### Composite resin group

The composite resin restorations accounted for the largest proportion of all the restorations assessed (57.9%). The composite resin restorations are divided into three categories: direct, indirect, or a combination of the two. The majority of all composite restorations were placed in a direct manner and predominantly on anterior teeth, evenly distributed between the maxilla and the mandible (Table 6).

**Table 6:** Breakdown of restoration types within composite resin group

	Frequency	Percent
Direct composite resin	210	70.5
Direct + indirect composite resin	81	27.2
Indirect composite resin comp	7	2.3
**Total**	**298**	**100.0**

#### Cast restorations group

The 172 cast restorations represented 32.6% of the restorations originally placed. A wide variety of restorations were included in this group. The majority of cast restorations were either metal-ceramic crowns, full gold crowns or adhesive gold onlays. These were predominantly placed on posterior teeth (Table 7).

**Table 7:** Breakdown of restoration types within cast restoration group

	Frequency	Percent
Metal-ceramic crown	65	37.8
Full gold crown	24	13.9
3/4 gold crown	12	7.0
Gold onlay	43	25
Ceramic onlay	2	1.2
Ceramic crown	2	1.2
Tooth-supported FPD	24	13.9
**Total**	**172**	**100.0**

#### Implant restorations group

Implant restorations accounted for 6.8% of all restorations placed. This included single and multiple units (Table 8).

**Table 8:** Breakdown of restoration types within implant restorations group

	Frequency	Percent
Implant single restoration	23	63.9
Implant FPD	13	36.1
**Total**	**36**	**100.0**

#### Amalgam restorations group

Amalgam restoration accounted for 4.0% of all restorations placed.

### Failure of restorations

In total, 66 out of the 527 teeth (12.5%) suffered major failures. This included restorations that had subsequently been replaced and those that had failed but required replacement.

A total of 252 out of 575 restorations were categorised as minor failures. The majority of which were found to be in the composite resin group (67.1%). Minor failures that involved repair in the past were mainly due to restoration fracture - 77.3% of all major failures were within this same group. Within the cast restoration group, 63.4% of restorations were deemed to be successful, free of any complications. (Table 9).

**Table 9:** Breakdown of combined failures within restoration groups

		No failure	Minor failure	Major failure	Total
Composite resin	Count	78	169	51	298
	% within composite resin group	26.2%	56.7%	17.1%	100.0%
	% within failure group	37.3%	67.1%	77.3%	56.5%
	% of total	14.8%	32.1%	9.7%	56.5%
Cast restorations	Count	109	54	9	172
	% within cast restoration group	63.4%	31.4%	5.2%	100.0%
	% within failure group	52.2%	21.4%	13.6%	32.6%
	% of total	20.7%	10.2%	1.7%	32.6%
Implant restorations	Count	17	17	2	36
	% within implant restoration group	47.2%	47.2%	5.6%	100.0%
	% within failure group	8.1%	6.7%	3.0%	6.8%
	% of total	3.2%	3.2%	0.4%	6.8%
Amalgam	Count	5	12	4	21
	% within amalgam group	23.8%	57.1%	19.0%	100.0%
	% within failure group	2.4%	4.8%	6.1%	4.0%
	% of total	0.9%	2.3%	0.8%	4.0%
	Count	209	252	66	527
	% within all restoration groups	39.7%	47.8%	12.5%	100.0%
	% within failure group	100.0%	100.0%	100.0%	100.0%
	% of total	39.7%	47.8%	12.5%	100.0%

When considering combined major and minor failures of each group, the majority of the restorations failed as a result of wear (21.2%), marginal discolouration (16.3%), surface roughness and marginal adaptation (Table 9).

### Survival analysis

**Fig. 2:**
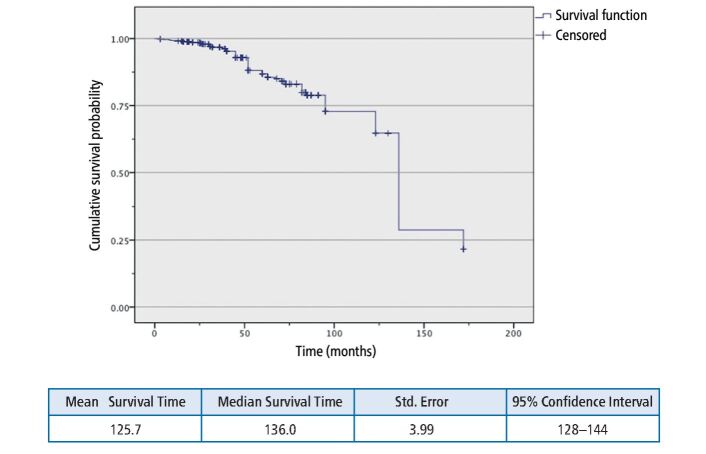
Kaplan-Meier survival plot for major failures in all restorations

**Fig. 3:**
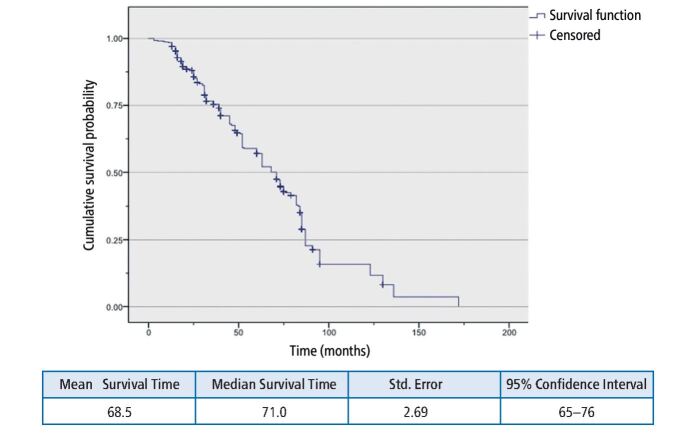
Kaplan-Meier plot for major and minor failures in all restorations

The median survival time (MST) for all the restorations was 136 months (11.3 years) when considering major failure modes only (Table 10, Fig. 2). This means that considering the group, there was a 50% probability of all restorations placed using this regimen to survive 11.3 years. However, this is when all restorations are pooled together. At combined major and minor failure levels, the MST was 71 months (5.92 years) (Table 11, Fig. 3).

**Table 10:** MST for major failures - restoration categories

	Total N	N of events	Mean survival time	Median survival time
Composite resin	298	51	115.969	136.000
Cast restoration	172	9	117.476	-
Implant restoration	36	2	123.757	-
Amalgam	21	4	74.395	-
Overall	527	66	125.725	136.000

**Table 11:** MST for combined failures - restoration categories

	Total N	N of Events	Mean survival time	Median survival time
Composite resin	298	220	59.915	52.000
Cast restoration	172	63	81.646	85.000
Implant restoration	36	19	90.672	75.000
Amalgam	21	16	58.660	63.000
Overall	527	318	69.761	71.000

Further statistical analysis involving log rank tests and Cox proportional hazards model revealed aetiology, restoration type, incisor relationship and tooth location as significant variables affecting survival rates when considering major failures (Table 12). Time to major failure was lower in Class II Div 2, lower arch and anterior teeth. Attrition, Class II Div 2 and Class III incisor relationships were identified to be significant. Material choice was significant when selecting composite resin or amalgam. Finally, lack of provision of occlusal splints appear to have a significant impact on time to failure with only six of the 18 patients wearing splints when assessed.

**Table 12:** Modes of failure

Cause of failure	Minor failure (% off all 527 restorations)		Major failure (% of all 527 restorations)		Combined failure (% of all 527 restorations)	
Fracture	20	3.8%	8	1.5%	28	5.3%
Loss of retention	5	0.9%	3	0.6%	8	1.5%
Caries	2	0.4%	3	0.6%	5	0.9%
Endodontic	-	-	6	1.1%	6	1.1%
Tooth Fracture	-	-	7	1.3%	7	1.3%
Anatomic form	49	0.9%	17	3.2%	66	12.5%
Marginal adaptation	76	14.4%	9	1.7%	85	16.1%
Wear	92	17.5%	20	3.8%	112	21.2%
Surface roughness	82	15.6%	5	0.9%	85	16.1%
Marginal discolouration	78	14.8%	8	1.5%	86	16.3%
Colour match	64	12.1%	2	0.4%	66	12.5%
Post-operative pain	5	0.9%	4	0.8%	9	1.7%
Gingival health	34	6.4%	1	0.2%	35	6.64%

When considering all forms of failure, increased age, women, attrition, Class III incisor relationship, lower arch, anterior teeth, plastic restorations and lack of splint were all significantly associated with an increased risk of any failure (Table 13).

**Table 13:** Significant clinical variables affecting survival outcome (p <0.05)

Variable	Log Rank P	Breslow P	Tarone-Ware P
Sex	0.060	0.009	0.013
Aetiology	0.000	0.000	0.000
Restoration type	0.001	0.010	0.002
Incisor relationship	0.000	0.000	0.000
Anterior/posterior	0.000	0.006	0.001
Maxilla/mandible	0.010	0.001	0.001
Opposing dentition	0.333	0.560	0.481
Post-op splint worn	0.068	0.471	0.228

### Biological complications

Only six restorations during pre-op required root canal treatment and five teeth in total were diagnosed with caries at some point following completion of treatment. Five patients received endodontic treatment from their general dental practitioners after developing symptoms, whilst one patient received root canal treatment mid-way through crown preparation with their own general dental practitioner. Radiographic examination was not carried out in this study and no patients showed signs of pulpal pathology.

### Patient satisfaction

The 21 major failures occurred in nine of the patients reviewed. Of these, two patients had tooth extractions, three patients received endodontic treatment and two patients had cast restorations provided on one or more teeth.

#### Results from OHRQoL-UK questionnaire

**Fig. 4:**
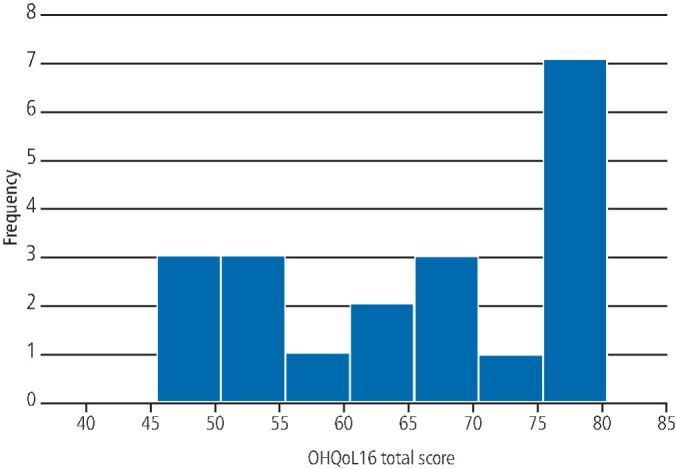
Histogram of OHRQoL scores distribution for all patients

The mean OHRQoL score reported for the 20 participants was 65.5. The minimum reported score was 48 and the maximum reported score was 80 (Fig. 4). This represents a positive benefit to the treatment provided.

#### Satisfaction with treatment at EDH

The participants were asked to report their satisfaction with four different aspects of their treatment. Overall satisfaction was high (Table 14).

**Table 14:** Descriptive results of participants' satisfaction with treatment

Satisfaction with	Satisfied	Indifferent	Dissatisfied
Improvement in dental condition	20 (100%)	0 (0%)	0 (0%)
Treatment received at EDH	20 (100%)	0 (0%)	0 (0%)
Duration of treatment at EDH	16 (80%)	1 (5%)	3 (15%)
Aesthetics following treatment	18 (90%)	2 (10%)	0 (0%)

## Discussion

This study shows that the use of anterior composite resin restorations combined with posterior cast or indirect restorations is a viable mid- to long-term treatment option to treat generalised tooth wear at an arbitrarily increased OVD. The OVD was selected in each case to provide ideal appearance and adequate thickness of restorative material. However, more than 60% of all the restorations exhibited some form of failure (minor or major) and required intervention and maintenance. Of all failures, the vast majority (69%) occurred within the composite resin restorations due to fracture, wear and marginal discolouration. These commonly occurred in combination.

The overall complication rate within the extra coronal restoration group was low. Around 63% of these restorations were deemed to be successful. This low complication rate is in keeping with findings of previous studies on survival of crowns^8^ and compare well to those placed within the general dental services in the UK.^9^ Cast gold restorations historically have been reported to have high survival rates;^10^ however, success is less frequently reported. The complication rate in this study appears to be higher than those reported for tooth borne prostheses in a systematic review by Pjetursson *et al.*^11^ Given the cohort of patients selected for this study being treated for tooth wear, the difference in overall complications should be expected. However, some similarities were found with Pjetursson *et al.*^11^ There appeared to be a greater proportion of implant restoration suffering complications than extra coronal restorations. This is likely due to the differences in the way forces are distributed between implant and tooth borne restorations in parafunctional patients. The periodontal ligament allows for a certain amount of shock-absorbing capacity that implant restorations do not have, which may explain the higher rates of technical complications in implant restorations.

At the major failure level requiring replacement of the restorations, the MST for all restorations pooled together was 136 months (11.33 years). For all failures (combined major and minor), the MST for all restorations was 71 months (5.9 years). A similar study by Smales and Berekally^12^ found ten-year cumulative survival estimates were 62% for composite resin restorations and 74.5% for all indirect restorations. When considering major failures, the present study found a cumulative survival rate at the ten-year mark of around 52.0% for composite resin restorations and 86.0% for cast restorations. However, given the limited number of patients presenting with treatment carried out over ten years ago, the results may not be truly representative. A better comparison would be the five-year survival rates, which appear to be similar to those of Smales and Berekally.^12^ Cumulative survivals for composite resin and cast restorations were found to be in order of 78% and 84%, respectively, whereas, the present study found survivals of 78% and 96%, respectively.

In a study by Gulamali *et al.,*^7^ composite resin restorations placed in cases with localised anterior tooth wear were reported to have MST of seven years for major failures. The MST for the composite resin group in the present study appears to be an improvement on those reported by Gulamali *et al.*^7^ when assessing major failures. However, it is lower when comparing combined major and minor failures within the composite resin group of the present study (52 months as opposed to 69 months). This may be attributed to more severe cases being treated with potentially greater demands on the restorative material.

The prospective study by Milosevic and Burnside^13^ assessed the survival of composite resin restorations placed in patients with severe tooth wear. Drawing comparisons with this study remain difficult since the restorations assessed were predominantly on anterior teeth and failure determination methods were not clearly defined. The annual failure rate in the first year was estimated to be 5.4%. The present study found an estimated annual failure rate of 2% and 17% when considering major or combined major and minor failures, respectively. Interestingly, Milosevic and Burnside^13^ did find that a lack of posterior support was a significant factor associated with failure. This is in keeping with the findings of the present study and may offer some explanation for the increased survival in comparison with the study of Gulamali *et al.*^7^

At both levels of failure, major and combined major and minor patients with attrition as the primary aetiological factor had restorations with significantly lower survival outcome when compared to erosion or combined aetiology. However, primarily attrition was diagnosed in only four patients, and it was not possible to establish one clear aetiological factor in those subjects diagnosed with combined aetiology. These results should be interpreted cautiously given the disproportionate representation of different categories within the aetiological variables and large amount of right censoring.

No analysis was carried out between the different types of composite resin or between direct and indirect approaches. Very few studies compare the use of direct and indirect restorations in the management of tooth wear. In this study, 70.5% of the composite restorations were placed directly leaving insufficient numbers to compare techniques. The statistical analysis in the present study suggested that for both major and combined failures, indirect cast restorations fared significantly better than composite resin restorations. It was not possible to determine a median survival time for cast restorations since only nine terminal events occurred through a ten-year period. Most restorations were without failures at the time of assessment thus giving an estimated cumulative survival of between 74-94%. Comparable results were reported by Smales and Berekally,^12^ with a ten-year cumulative survival of 74.5% for indirect cast restorations. The authors did also suggest a strong trend for lower survivals in composite resin restorations, but, unlike the present study, did not find any statistical significance due to the very small numbers involved.

The nature of the incisal relationships has been reported to have an impact on the survival of restorations placed in severe tooth wear. Milosevic and Burnside^13^ found a greater proportion of failures in Class III incisal relationship but was not statistically significant. Conversely, *Gulamali et al.*^7^ found there was a statistically significant, better outcome in patients with Class III incisor relationships, at both levels of failure assessment. The present study did find a statistically significantly difference in survival for different incisor relationships. Patients presenting with a Class III incisor relationship had a greater proportion of both major and minor failures. Possibly unfavourable, heavier shear and tensile forces on the restorative material associated with a Class III occlusion could explain such a finding.

This finding should be taken with caution given that only one patient represented the Class III category. The log rank test does not allow to test the effect of the other independent variables or account for clustering of restorations within patients.

Position of the tooth within the arch may contribute to potential failures. The nature of forces exerted on a tooth vary depending on the position and the occlusal scheme. Restorations on anterior teeth, depending on the clinical appearance of the wear, may be placed under significant shear stresses.

In the present study, restorations placed on anterior teeth or in the mandibular arch presented with lower median survival times for combined major and minor failure. Lower anterior teeth tend to offer reduced surface area for bonding which may explain these findings.

Studies assessing the role of tooth position have suggested that a greater proportion of failures appear to occur on anterior teeth and the lower arch. Smales and Berekally^12^ suggested this difference might be the result of more restorations on anterior teeth. Milosevic and Burnside^13^ demonstrated a higher proportion of failures in the mandible, but no statistical significance was found. The present study appears to reflect the findings of these two studies. Another study by Al-Khayatt *et al.*^14^ reported specifically on survival of restorations on lower anterior teeth. The authors found survival rate of 85% at seven years; however, a small sample size and lack of adjustment of statistical analysis to allow clustering of restorations in patient may have influenced findings.

Occlusal stabilisation splints were provided for 18 out of the 20 subjects, including all those diagnosed with attrition, were provided with occlusal stabilisation splints following completion of treatment. Despite this, compliance appeared to be poor, with only six subjects still wearing the splint at the time of assessment. The majority of patients reported diminishing compliance with splint wearing over the first 24-36 months.

When assessing major and minor failures combined, greater survival outcomes were observed in those patients wearing post treatment splints. The protective barrier offered by a splint may also have contributed to a reduction in minor failures in the compliant patient group. More compliant patients may also take greater care of their teeth and restorations with respect to plaque control.

The overall mean OHRQoL scores reported by participants in this study were greater than those of the British public norms.^15^ However, the sample was too small for separation into age categories or for any statistical analysis, therefore not allowing further interpretation of these results.

Assessment of participants' satisfaction was carried out in four further questions. Since these questions had not been validated, they were not included in the total score reported; however, they do provide valuable information on the management of such cases.

All participants reported general improvement in their dental condition. Since the treatment lead to improvement in the patient function and appearance, this finding is as expected. Satisfaction with the treatment received was also 100%. Most subjects were happy with the dental aesthetics on completion of treatment; however, two were indifferent. These were mainly related to colour matching and discoloration of composite restorations.

The greatest level of dissatisfaction came from the duration of treatment, where three participants reported dissatisfaction. The average treatment time was 36 months. The three dissatisfied patients had treatment times over 71 months on average with treatment re-allocation to new postgraduates involved.

## Conclusion

Within the limitations of this retrospective study, the following conclusions can be drawn:

- The management of tooth wear with anterior composite resin restorations with posterior cast or indirect restorations is a viable mid to long term treatment option. Median survival times for all restorations when considering major failures was 11.3 years
- A relatively small number of major complications were observed, and most were attributed to mechanical failures, with very few biological complications observed
- Composite resin restorations are susceptible to minor failures requiring some maintenance
- There was a statistically significant difference between the survival of composite resin restorations and indirect or cast restorations
- Factors associated with failure were location of restorations on anterior teeth, in the mandible and in cases with attrition
- Occlusal splints were made for 18 of the 20 patients but at the time of assessment only six patients were wearing them
- High overall satisfaction scores were recorded. Greatest dissatisfaction was associated with duration of treatment.

## Data Availability

The data that support the findings of this study are available from the corresponding author upon reasonable request.
